# Compensatory *trans*‐regulatory alleles minimizing variation in *TDH3* expression are common within *Saccharomyces cerevisiae*

**DOI:** 10.1002/evl3.137

**Published:** 2019-08-29

**Authors:** Brian P. H. Metzger, Patricia J. Wittkopp

**Affiliations:** ^1^ Department of Ecology and Evolutionary Biology University of Michigan Ann Arbor Michigan 48109; ^2^ Department of Ecology and Evolution University of Chicago Chicago Illinois 60637; ^3^ Department of Molecular, Cellular, and Developmental Biology University of Michigan Ann Arbor Michigan 48109

**Keywords:** Compensation, eQTL, gene regulation, mapping, stabilizing selection

## Abstract

Heritable variation in gene expression is common within species. Much of this variation is due to genetic differences outside of the gene with altered expression and is *trans*‐acting. This *trans*‐regulatory variation is often polygenic, with individual variants typically having small effects, making the genetic architecture and evolution of *trans*‐regulatory variation challenging to study. Consequently, key questions about *trans*‐regulatory variation remain, including the variability of *trans*‐regulatory variation within a species, how selection affects *trans*‐regulatory variation, and how *trans*‐regulatory variants are distributed throughout the genome and within a species. To address these questions, we isolated and measured *trans*‐regulatory differences affecting *TDH3* promoter activity among 56 strains of *Saccharomyces cerevisiae*, finding that *trans*‐regulatory backgrounds varied approximately twofold in their effects on *TDH3* promoter activity. Comparing this variation to neutral models of *trans*‐regulatory evolution based on empirical measures of mutational effects revealed that despite this variability in the effects of *trans*‐regulatory backgrounds, stabilizing selection has constrained *trans‐*regulatory differences within this species. Using a powerful quantitative trait locus mapping method, we identified ∼100 *trans*‐acting expression quantitative trait locus in each of three crosses to a common reference strain, indicating that regulatory variation is more polygenic than previous studies have suggested. Loci altering expression were located throughout the genome, and many loci were strain specific. This distribution and prevalence of alleles is consistent with recent theories about the genetic architecture of complex traits. In all mapping experiments, the nonreference strain alleles increased and decreased *TDH3* promoter activity with similar frequencies, suggesting that stabilizing selection maintained many *trans*‐acting variants with opposing effects. This variation may provide the raw material for compensatory evolution and larger scale regulatory rewiring observed in developmental systems drift among species.

**Impact Summary**

Gene expression varies among individuals in a population due to genetic differences in regulatory components. To determine how this variation is distributed within genomes and species, we used a powerful genetic mapping approach to examine multiple strains of *Saccharomyces cerevisiae*. Despite evidence of stabilizing selection maintaining gene expression levels among strains, we find hundreds of loci that affect expression of a single gene. These loci vary among strains and include similar frequencies of alleles that increase and decrease expression. As a result, each strain contains a unique set of compensatory alleles that lead to similar levels of gene expression among strains. This regulatory variation might form the basis for large scale regulatory rewiring observed among distantly related species.

Heritable variation in gene expression results from genetic variation affecting *cis*‐regulatory elements (e.g., promoters and enhancers) and *trans*‐acting factors (e.g., proteins and RNAs). These *trans*‐regulatory changes are located throughout the genome and are the major source of regulatory variation within species (Wittkopp et al. 2004; Wang et al. 2007; Sung et al. 2009; Zhang and Borevitz 2009; Emerson et al. 2010; Bell et al. 2013; Schaefke et al. 2013; Suvorov et al. 2013; Coolon et al. 2014; Chen et al. 2015). The number, identity, and effects of individual loci contributing to variation in gene expression have been determined in a variety of species using expression quantitative trait locus (eQTL) mapping (Gilad et al. 2008; Hansen et al. 2008; Majewski and Pastinen 2011; Cubillos et al. 2012; Nica and Dermitzakis 2013; Westra and Franke 2014; Albert and Kruglyak 2015; Pai et al. 2015), with the most extensive dissection of eQTL coming from studies of two strains of the baker's yeast *Saccharomyces cerevisiae* (Brem et al. 2002, 2005; Schadt et al. 2003; Yvert et al. 2003; Brem and Kruglyak 2005; Ronald et al. 2005; Smith and Kruglyak 2008; Albert et al. 2014, 2018; Parts et al. 2014). These studies have found that (1) expression differences are typically associated with ∼10 or fewer eQTL, (2) most eQTL have individually small effects on expression, and (3) most eQTL do not overlap the gene whose expression they affect and thus likely contribute to *trans*‐regulatory differences.

Traditional eQTL mapping approaches require genotype and expression data for many individuals to detect significant effects. Consequently, studies mapping the genetic basis of regulatory differences have largely been limited to two strains within any given species. In cases where the extent and variability of regulatory variation have been studied within a species, experiments have focused on *cis*‐regulatory variation for technical reasons (de Meaux 2005; Gruber and Long 2009; Kang et al. 2016; Moyerbrailean et al. 2016; Salinas et al. 2016; Kita et al. 2017). As a result, key questions about the extent, variability, and genetic basis of *trans*‐regulatory variation segregating within a species remain unanswered. For example, do multiple *trans*‐regulatory variants affecting a gene's expression often segregate at the same locus within a species? How different are the suites of *trans*‐acting eQTL affecting a gene's expression among individuals or strains? Are the effects of *trans*‐regulatory variants at different loci often in the same direction, or do they typically have opposing effects, canceling one another out? Addressing these questions requires identifying *trans*‐acting eQTL and their effects on expression among multiple individuals or strains of the same species. Although genome‐wide association studies use population level variation to identify eQTL, they do not meet this need because they can only detect eQTL alleles that are shared by many individuals.

In addition to these questions about the variability in genetic architecture of *trans*‐regulatory variation, questions also remain about the impact of selection on this variation. Prior work has shown that gene expression levels are broadly constrained by stabilizing selection (Denver et al. 2005; Gilad et al. 2006) and variation in *cis*‐regulatory eQTL appears to be limited by purifying selection (Josephs et al. 2015; Kita et al. 2017). But the impact of natural selection on the number, identity, or genomic distribution of *trans*‐acting eQTL is less clear, and there are reasons to suspect that it might be different than for *cis*‐acting eQTL. For example, prior work suggests that *trans*‐regulatory mutations arise more frequently than *cis*‐regulatory mutations, but tend to have smaller effects on the focal gene's expression (Metzger et al. 2016). In addition, *trans*‐regulatory mutations are more likely to be recessive and have greater pleiotropic effects than *cis*‐regulatory mutations (Stern 2000; Landry et al. 2007; Fay and Wittkopp 2008; Lemos et al. 2008; Gruber et al. 2012). Any or all of these factors might cause selection for the level of gene expression to have different impacts on *cis*‐ and *trans‐*regulatory variation.

Here, we examine *trans*‐regulatory variation segregating among genetically distinct strains of *S. cerevisiae*. We focus on the extent and variability of, evolutionary forces acting on, and genetic basis for, *trans*‐regulatory variation affecting expression of the *TDH3* gene, which encodes a glyceraldehyde‐3‐phosphate dehydrogenase. This gene was chosen because prior work has estimated the effects of new *trans*‐regulatory mutations on its expression (Gruber et al. 2012; Metzger et al. 2016) as well as the fitness consequences of changing its expression (Duveau et al. 2017, 2018), allowing us to compare the *trans*‐regulatory variation segregating in *S. cerevisiae* to empirically informed models of neutral evolution. We find that although differences in *trans*‐regulation affecting *TDH3* promoter activity are common among strains, they generate less variation in *TDH3* promoter activity than predicted by neutral models, suggesting that stabilizing selection has acted on *trans*‐regulatory variation affecting *TDH3* promoter activity in the wild. We then use a powerful genetic mapping approach to determine differences in the genetic architecture of this *trans*‐regulatory variation by identifying eQTL between each of three strains of *S. cerevisiae* and a common reference strain. In each of these three eQTL mapping experiments, we find an order of magnitude more eQTL affecting activity of the *TDH3* promoter in *trans* than previously known. These loci are often different among strains, have opposing effects on expression, and are spread throughout the genome, indicating diverse sources of *trans*‐regulatory variation segregating within *S. cerevisiae*. These results agree with theoretical predictions that stabilizing selection can maintain genetic variation for polygenic traits (Lande 1976; Dover and Flavell 1984; Turelli 1984; Barton 1986, 1989). They also suggest that natural populations harbor greater regulatory variation than suggested by differences between a single pair of strains and that this variation can impact the evolution of regulatory systems.

## 1 Results and Discussion

**Figure 1:**
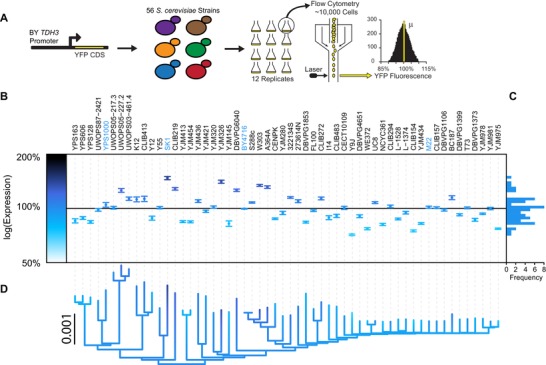
Extensive *trans*‐regulatory variation affecting *TDH3* expression is segregating among *S. cerevisiae* strains. (A) Variation in *TDH3 trans*‐regulatory backgrounds among yeast strains was measured using a reporter gene containing the *TDH3* promoter from the BY strain and a yellow fluorescent protein (YFP). This reporter was integrated into the genome of 56 diverse *S. cerevisiae* strains. Twelve replicate populations were grown in YPD and analyzed by flow cytometry for YFP expression. (B) Variation among replicates relative to the BY reference strain was used to calculate the average effect of each strain's *trans*‐regulatory background on *TDH3* promoter activity. Darker colors reflect higher *TDH3* reporter activity. Strain names in blue are used in subsequent mapping experiments. (C) Frequency of *trans*‐regulatory effects relative to reference strain. (D) Phylogenetic relationships among strains as estimated from genome‐wide polymorphism data (MacLean et al. 2017). Color of branches corresponds to estimated *trans*‐regulatory effect from ancestral character estimation.

To isolate the effects of *trans*‐regulatory variants segregating among *S. cerevisiae* strains on *TDH3* promoter activity, we inserted a yellow fluorescent protein (YFP) coding sequence under control of the 678bp *TDH3* promoter allele from the BY lab strain into 56 distinct *S. cerevisiae* strains (Fig. 1A). These strains (1) were isolated from a range of environments, (2) differ at more than 100,000 SNPs and small indels, many larger copy number variants chromosomal rearrangements, and (3) encompass much of the genetic and phenotypic diversity observed within the species (MacLean et al. 2017; Peter et al. 2018). For each strain, we measured YFP fluorescence in 12 biological replicate populations grown in rich media and used the measured YFP fluorescence to estimate changes in *TDH3* mRNA levels due to differences in *trans*‐regulation among strains (Duveau et al. 2018). We observed that *trans*‐regulatory variation caused differences in expression that ranged from 71% to 147% of the reference strain (Fig. 1B and 1C). This variability in *trans*‐regulation was nearly double the variability of *cis*‐regulation described in a previous study among a similar set of strains (Fig. S1A; Metzger et al. 2015). We detected significant phylogenetic structure for *trans*‐regulatory differences among strains, with more closely related strains having on average more similar *TDH3* promoter activity than more distantly related strains (*λ* = 0.59, *P* = 0.013; *K* = 0.49, *P* = 0.012; Fig. 1D).

**Figure 2:**
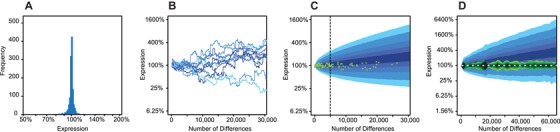
Natural selection has constrained *TDH3 trans*‐regulatory variation. (A) Effects of *trans*‐regulatory mutations on *TDH3* promoter activity. Mutants were collected and analyzed in prior work (Metzger et al. 2016). (B) Simulated neutral trajectories for *TDH3* promoter activity based on empirically measured effects of new mutations. Lighter colors reflect more extreme values after 30,000 mutations. (C) Comparison of observed differences in *TDH3* promoter activity among *S. cerevisiae* strains with neutral expectation. The blue background represents the 95th, 90th, 80th, 70th, and 60th percentiles, from light to dark, for the simulated neutral trajectories. Green dots are differences in *TDH3* promoter activity and estimated number of mutations based on the *S. cerevisiae* phylogeny. Dashed line indicates the point where the observed data depart significantly from expectation. (D) Same as (C), but using genetic distance instead of phylogenetic distance among strains. The green areas represent the 95th, 90th, 80th, 70th, and 60th percentiles, from light to dark, for the observed differences from sampling pairs of strains.

In the absence of natural selection, the effects on expression of naturally occurring regulatory variation should be similar to the effects on expression of new mutations. Differences between the effects of naturally occurring variation and new mutations thus provide evidence of natural selection (Metzger et al. 2015). To determine whether natural selection has impacted *trans*‐regulatory variation affecting *TDH3* promoter activity, we constructed models of neutral evolution using the effects of new mutations defined in prior work (Metzger et al. 2016). These mutations were generated with the mutagen EMS (Metzger et al. 2016), which mimics the most common type of point mutation in yeast (Zhu et al. 2014) and the most common type of polymorphism found among natural yeast strains (G→A and C→T; MacLean et al. 2017). We simulated the neutral evolution of *trans‐*regulatory variation affecting *TDH3* promoter activity by sampling these *trans*‐regulatory mutational effects on *TDH3* promoter activity (Fig. 2A) and tracking how expression changed with the addition of each new mutation, assuming additivity (Fig. 2B). We repeated this sampling process 10,000 times and used the observed distributions of expression levels after the addition of each new mutation to define the probability with which we expect to see a given expression level evolve neutrally from the common ancestor after a particular number of genetic changes. Epistasis was ignored in this simulation because its prevalence and effects are unknown for these mutations; it could either increase or decrease the range of expression differences arising from mutation alone. (For a more complete discussion of modeling assumptions and rationale, see the Supporting Information Methods section).

We compared our neutral projections to the observed differences in *TDH3* promoter activity among strains, using the genetic relationships among strains to infer how *TDH3* promoter activity changed along each branch of the phylogeny (Fig. 2C). We found that there was significantly less *trans*‐regulatory variability among strains than predicted to arise from mutation alone, suggesting that natural selection has constrained *TDH3* promoter activity (*P* < 0.0001; Fig. S1B and S1C). Indel mutations were not included in our distribution of mutational effects but are likely contributing to expression differences among strains; however, these mutations are expected to cause even larger deviations in gene expression than point mutations, further increasing the variation in gene expression expected under neutrality and making our conclusion conservative. To determine whether our inference of stabilizing selection was robust to uncertainty in the inferred phylogenetic relationships among strains and the inferences of changes in *TDH3* promoter activity on the phylogeny, we repeated this analysis using the total genetic distance between pairs of strains instead of the phylogenetic relationships among strains. We again found less *trans*‐regulatory variability in *TDH3* promoter activity among strains than predicted by the neutral model, further supporting the hypothesis that *trans*‐regulatory variation affecting *TDH3* promoter activity has evolved under stabilizing selection (Fig. 2D; Fig. S1D).

We also tested for evidence of natural selection acting on *TDH3 trans*‐regulatory variation using a more traditional approach that does not rely on empirical estimates of the effects of new *trans*‐regulatory mutations. Specifically, we fit the *P_TDH3_*‐YFP reporter activity and phylogenetic relationships among strains to two models of quantitative trait evolution: a Brownian motion model of neutral quantitative trait evolution and an Ornstein–Ulenbeck model that incorporates stabilizing selection (Bedford and Hartl 2009). We found that the Ornstein–Ulenbeck model fit the data significantly better than the neutral Brownian motion model (*P* = 0.00007, chi‐square test; Fig. S1E and S1F), again suggesting that *trans*‐regulatory variation affecting *TDH3* promoter activity in *S. cerevisiae* has been shaped by stabilizing selection. The magnitude of effects of *trans*‐regulatory variation on *TDH3* promoter activity is expected to decrease fitness by less than 0.1% for 80% of strains based on prior work describing the relationship between *TDH3* expression level and fitness in rich media (Duveau et al. 2018), with the largest deviation in *TDH3* promoter activity (71% of wild type) expected to decrease fitness by 0.5% (Fig. S1G). Given the large effective population size of *S. cerevisiae*, we conclude that weak stabilizing selection has constrained the *trans*‐regulatory evolution of *TDH3*, although we cannot rule out that selection acting on pleiotropic effects of these regulatory variants might also have contributed to this signal.

**Figure 3:**
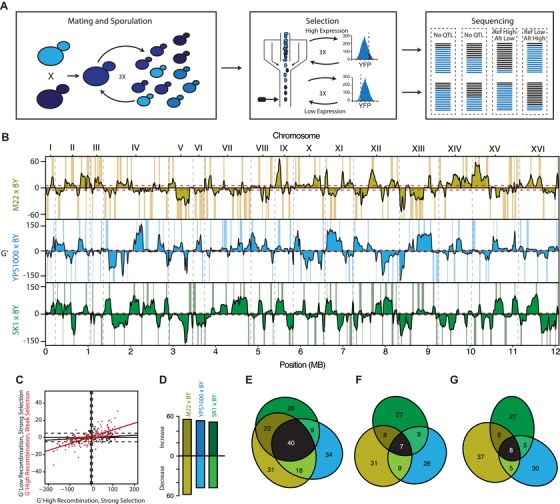
Compensatory alleles underlie the maintenance of *TDH3 trans*‐regulatory effects. (A) The genomic basis of *TDH3 trans*‐regulatory variation was mapped using an xQTL approach. Left: Three rounds of mating and sporulation were used to increase mapping resolution. Middle: Three rounds of FACS based selection were used to enrich for alleles increasing and decreasing *TDH3 trans*‐regulatory activity. In each round, the top or bottom 5% of the population was collected. Right: Comparisons of allele frequency from Illumina sequencing of FACS‐based pools was used to identify eQTL. Each block (dashed lines) represents a different genomic region. Colored lines represent allele frequencies. Black: Reference strain. Blue: Testing strain. For each block, the top bars are after selection for high YFP fluorescence, while the bottom bars are after selection for low YFP fluorescence. eQTLs are identified when allele frequencies among the high and low selected pools differ significantly. (B) G’ statistic for evidence of eQTL in each comparison. Effects are relative to the non‐BY reference allele. Dashed gray lines indicate chromosome boundaries. Dashed red lines gives threshold for statistical significance. Called eQTLs with 95% confidence intervals on the location are highlighted for each strain. Brown: M22 × BY. Blue: YPS1000 × BY. Green: SK1 × BY. (C) Relationship between G’ statistic for different mapping procedures. X‐axis—G’ statistic for high recombination and strong selection (three rounds of crossing and three rounds of selection). Y‐axis—(Black) G’ statistic for low recombination and strong selection (one round of crossing and three rounds of selection). (Red) G’ statistic for high recombination and weak selection (three rounds of crossing and one round of selection). Each point is for an eQTL identified with high recombination and strong selection (three rounds of crossing and three rounds of selection) from the M22 × BY cross. Solid lines show fits from a linear model for high recombination and low selection (red) or low recombination and high selection (black). (D) Number of non‐BY eQTL increasing or decreasing *TDH3* promoter activity for each cross. (E) eQTL shared among the three crosses irrespective of direction of effect. Areas are proportional to the number of eQTL shared. Brown: eQTL identified only in the M22 × BY cross. Blue: eQTL identified only in the YPS1000 × BY cross. Green: eQTL identified only in the SK1 × BY cross. Black: eQTL identified in all three crosses. (F) Same as (E), but for non‐BY eQTL that increase *TDH3* promoter activity. (G) Same as (E), but for non‐BY eQTL that decrease *TDH3* promoter activity.

In the presence of stabilizing selection, gene expression can be kept similar among strains by purging mutations that alter expression or by maintaining sets of variants with off‐setting, or compensatory, effects on expression in the population. To determine which of these mechanisms is more likely to have minimized differences among strains in the *trans*‐regulatory effects on *TDH3* promoter activity, we used eQTL mapping to examine the genetic architecture of *trans*‐regulatory variation affecting *TDH3* promoter activity in three strains (M22, YPS1000, and SK1) relative to a common reference strain (BY). These strains differ in the effects of their *trans*‐regulatory background on *TDH3* promoter activity (from 101% to 147%) as well as their phylogenetic relatedness (Fig. S2A), making them ideal for determining how the genetic architecture of *TDH3 trans*‐regulation varies within the species (Fig. S2A). For each focal strain, we used extreme QTL mapping with three rounds of crossing followed by three rounds of selection on *P_TDH3_*‐YFP expression using fluorescence‐assisted cell sorting (FACS; Fig. 3A; Ehrenreich et al. 2010, 2012; Kofler et al. 2011; Parts et al. 2011; Cubillos et al. 2013; Albert et al. 2014; Duveau et al. 2014; Schlötterer et al. 2014).

In all three crosses, we identified ∼100 eQTL at a false discovery rate of 10% (*n* = 113 for M22 vs. BY; *n* = 101 for YPS1000 vs. BY; *n* = 99 for SK1 vs. BY; Fig. 3B). This is approximately 10‐fold greater than the number of eQTL identified for most genes in a prior eQTL mapping study (Albert et al. 2018) between BY and another strain of *S. cerevisiae*, RM11, that is closely related to M22 (Fig. S2A), indicating considerable power to detect individual eQTL. The non‐BY alleles were evenly split between those that increased and decreased expression for all three mapping experiments (55 of 113 M22 alleles increase expression, 53 of 101 YPS1000 alleles increase expression, and 51 of 99 SK1 alleles increase expression, *P* > 0.6 for all, binomial test; Fig. 3D). This similarity in the frequency of eQTL increasing and decreasing expression could result from neutral evolution (assuming mutations increasing and decreasing *TDH3* expression arise with similar frequency) or stabilizing selection; however, the evidence of stabilizing selection described above suggests that similar *trans*‐regulatory effects on *TDH3* promoter activity are observed among strains of *S. cerevisiae* because compensatory alleles are maintained in the population. Consistent with this conclusion, we found that repeating these eQTL mapping experiments with crossing limited to a single round resulted in considerably fewer eQTL identified, regardless of the number of rounds of selection (black points in Fig. 3C; Fig. S3). By contrast, reducing the rounds of selection resulted in decreased statistical significance for many eQTL, but did not change the location or direction of effects for most eQTL inferred (red points in Fig. 3C; Fig. S3). These observations, which were robust to changing the statistical threshold used to call eQTLs (Tables S5 and S6), suggest that although additional rounds of selection allowed eQTL with smaller effects to be identified, the high number of eQTL detected results primarily from increased recombination during multiple rounds of meiosis breaking apart physically close eQTL with opposite effects on expression.

To determine the similarity in loci harboring *trans*‐regulatory variation affecting *TDH3* promoter activity among strains, we compared the genomic locations of eQTL identified in each pair of strains. If *trans*‐regulatory variation is caused by the same loci in all strains, the ∼100 eQTLs identified in each comparison should map to similar genomic regions. However, if the sources of *trans*‐regulatory variation affecting *TDH3* promoter activity segregating in *S. cerevisiae* are more diverse, eQTL identified in each comparison should map to different genomic regions. We found that the 313 eQTLs identified mapped to 180 nonoverlapping regions of the genome, with 27% (49 of 180) of these regions containing eQTL in only two of the comparisons and 22% (40 of 180) of these regions containing eQTL in all three comparisons (Fig. 3E). Such shared eQTL regions may contain genes that contribute to variation in *trans*‐regulation of *TDH3* promoter activity in multiple strains; however, the 49% of loci overlapping in at least two strains is not greater than expected by chance given the number and width of eQTL observed (*P* = 0.08, permutation test, 95% CI: 41–50%). Furthermore, in these shared genomic regions, only 18% of non‐BY eQTL alleles had the same direction of effect on *TDH3* promoter activity in two comparisons (26 of 119 for increases and 18 of 120 for decreases), and only 6% of non‐BY eQTL alleles had the same direction of effect in all three comparisons (seven of 119 for increases and eight of 120 for decreases; *P* > 0.53, permutation test; Fig. 3F–G). These results were robust to the FDR used for identifying peaks (Tables S7 and S8). This lack of consistency in the direction of eQTL effects suggests that even if the same underlying loci contribute to *trans*‐regulatory variation in multiple strains, the exact polymorphisms and their effects on *TDH3* promoter activity are likely to differ among strains.

To determine whether differences in the eQTL inferred among strains were more likely to be due to biological differences or poor reproducibility among independent experiments, we repeated the mapping experiment between M22 and BY and compared the eQTL identified in the two replicate mapping experiments (Table S9). Of the 74 eQTL found in the second M22/BY eQTL mapping experiment, 73% (54 eQTL) overlapped with eQTL from the initial M22/BY mapping experiment, which is significantly more than expected by chance (95% CI: 27–40%, *P* < 0.001, permutation test; Fig. S2C and S2D). This degree of overlap between the two M22/BY mapping experiments is also significantly greater than the degree of overlap between the second M22/BY experiment and the YPS1000/BY experiment (54%, 40 of 74 eQTL, *P* = 0.03, Fisher's exact test) but not the SK1/BY experiment (58%, 43 of 74 eQTL, *P* = 0.08, Fisher's exact test). Using a more stringent false discovery rate of 3% to identify eQTL reduced the number of eQTL called in the second M22/BY eQTL mapping experiment to 48, but there was still more overlap between the two M22/BY experiments than expected by chance (36 overlapping eQTL, 75%, 95% CI: 26–42%, *P* < 0.001, permutation test; Table S10). Overlap between the two M22xBY crosses was similar to the overlap between the second M22xBY eQTL mapping experiment and either of the other two crosses, however, suggesting that reducing the false discovery rate enriched for eQTL shared among all strains (Table S10). eQTLs identified between M22 and BY were also compared to eQTL identified previously between strains RM11 and BY. Consistent with the close phylogenetic relationship between M22 and RM11 (Fig. S2A), the 113 eQTL identified in the initial mapping between M22 and BY strains included all eight regions of the genome identified as affecting *TDH3* promoter activity in a prior cross between the BY and RM11 strains, seven of which had eQTL alleles with effects in the same direction (Albert et al. 2014; Fig. S2E). Six of these seven eQTLs with effects mapped in the same direction of the first M22 × BY cross and the previously published RM11 × BY crosses were also identified with the more stringent FDR cutoff of 3% (|G’|>10; Fig. 2E). In the second M22 × BY mapping experiment, we observed eQTL between M22 and BY with the same direction of effect as eQTL identified between RM11 and BY for four of the eight previously identified eQTLs (Fig. S2D). In all, more than 100 eQTLs have been identified for these closely related crosses, with the majority of eQTLs identified in two independent crosses. These results suggest that the differences in eQTLs mapped among strains are unlikely to be explained by low reproducibility of the mapping procedure, but rather reflect real differences in the genetic architecture of *trans*‐regulatory variation affecting *TDH3* promoter activity among strains.

Taken together, our data suggest that despite stabilizing selection limiting variation in *TDH3* expression, there are hundreds of genetic variants segregating within *S. cerevisiae* that impact *TDH3* promoter activity in *trans*. This number of variants is substantially more than suggested by prior work, indicating that *trans*‐regulatory variation is more polygenic than typically appreciated. These variants (1) differ among strains, (2) cause increases and decreases in *TDH3* promoter activity with similar frequencies, and (3) are located in hundreds of distinct regions in the genome. In the wild, even more loci are expected to harbor variation affecting *TDH3* expression because the shared reporter gene used in our experimental design eliminated variation in the *TDH3* promoter and was blind to variation affecting posttranscriptional regulation of *TDH3*. Our experimental design was also unable to determine interactions between *cis*‐ and *trans*‐regulatory variation, epistatic interactions among regulatory variants, the effect size of individual eQTL, or the specific SNPs responsible for each eQTL's effects. Nonetheless, the prevalence and distribution of loci we identified are similar to recent mapping experiments in *S. cerevisiae* that also had high power to detect loci with opposing effects on quantitative traits (Jakobson and Jarosz 2019). They are also consistent with models of complex traits, such as the “omnigenic model” (Boyle et al. 2017; Wray et al. 2018; Liu et al. 2019); our work specifically supports the idea that many casual variants have *trans*‐regulatory effects on expression.

Although it might seem counterintuitive to find such extensive genetic variation affecting a trait whose variance appears to have been limited by stabilizing selection, theoretical work has previously shown that stabilizing selection acting on quantitative traits can maintain abundant cryptic genetic variation with off‐setting effects (Lande 1976; Dover and Flavell 1984; Turelli 1984; Barton 1986, 1989). The pervasiveness of genetic variants with opposing effects on expression is consistent with the recurrent observation of compensatory evolution in genomic comparisons of gene expression within and among species (Goncalves et al. 2012; Schaefke et al. 2013; Coolon et al. 2014; Mack et al. 2016; Verta et al. 2016; Metzger et al. 2017). This variation may form the basis for developmental systems drift in which phenotypes stay stable over evolutionary time, but the molecular components responsible for the phenotype change (True and Haag 2001; Brachi et al. 2010; Gordon and Ruvinsky 2012; Pavlicev and Wagner 2012): with many combinations of alleles available that can produce the same trait value, changes in the regulation of a trait that do not alter the trait value might be common. Additional genetic mapping experiments that have similar power to resolve closely linked loci and detect alleles with very small effects are needed to determine whether the complex genetic architecture observed for the *trans*‐regulation of the *TDH3* gene in *S. cerevisiae* is common to other genes, traits, and organisms.

## 2 Methods

### 2.1 YEAST STRAINS AND GROWTH CONDITIONS

Strains used in this work are listed in Table S1. To determine variability in *TDH3* expression segregating within *S. cerevisiae*, we used haploid, MATalpha versions of 85 natural *S. cerevisiae* strains created in previous work (MacLean et al. 2017). For each strain, we inserted a P*_TDH3_*‐YFP reporter at the *HO* locus using a standard lithium acetate transformation approach with minor alterations (Cubillos et al. 2009; MacLean et al. 2017). The inserted reporter contained a copy of the 678 bp *TDH3* promoter from the BY strain (entire intergenic region 5’ of the *TDH3* coding sequence), a YFP coding sequence, a CYC1 terminator, and a NatMX4 drug resistance marker. For 60 strains, we obtained successful integration and correct sequence of the reporter. Prior work indicates that the location of the reporter, or the use of a fusion of YFP to native TDH3, has consistent effects on expression and we thus expect that this experiment primarily is reporting on *trans*‐regulatory variation among strains (Metzger et al. 2016). Unless noted, all yeast growth was performed at 30°C in YPD (1% Difco yeast extract, 2% peptone, and 2% glucose).

### 2.2 MEASUREMENT OF YFP EXPRESSION

The *trans*‐regulatory effects on *TDH3* promoter activity for each strain were estimated by measuring YFP expression from the P*_TDH3_*‐YFP reporter. Strains were first revived from glycerol stocks on YPG (1% Difco yeast extract, 2% peptone, and 2% glycerol) at 30°C. After 24 h, each strain was inoculated into liquid YPD in a 96‐well plate. For each plate, YFP positive (PJW1139) and YFP negative (PJW880) strains were included at specific locations in the 96‐well plate as controls. This structure was replicated to solid YPD using a pin tool. To generate replicates, colonies were pin‐tool replicated after 24 h into twelve 96‐well plates containing 500 µL of liquid YPD and grown for 24 h. Cultures were then diluted 1/20 into fresh 500 µL of YPD and grown for an additional 4 h. Samples were diluted 1/10 into 500 µL PBS and analyzed on an Accuri C6 flow cytometer connected to an Intellicyt autosampler.

Data were processed using the same procedure as described in Duveau et al. (2018). Briefly, hard gates were used to remove flow cytometry artifacts and instances where multiple cells entered the flow cytometry detector at the same time based on estimates of cell size. For each sample, the most abundant monomorphic population was identified and the effect of cell size on fluorescence removed. For each event in a sample, the YFP levels were converted to estimates of mRNA expression using the formula E(mRNA) = exp(–7.820027 × E[YFP]), which was based on a direct comparison of YFP fluorescence and mRNA abundance first reported in Duveau et al. (2018). From these estimates, the population median was calculated. Using the control strains, linear models were used to remove batch effects such as differences among plates and variation due to the position of a sample (row and column) within a plate. Twelve replicate samples from each strain were combined to estimate strain averages. Four strains—NCYC110 (PJW1041), EM93 (PJW1055), YIIc17_E5 (PJW1038), and DBVPG3591 (PJW1053)—were excluded from analysis due to inconsistent measurements among replicates caused by flocculation and cell settling.

The effects of naturally occurring *cis*‐regulatory variants on *TDH3* promoter activity within *S. cerevisiae* used data from Metzger et al. 2015 (Flow Repository FR‐FCM‐ZZBN). The effects of new mutations on *TDH3* promoter activity used data from Metzger et al. 2016 (Flow Repository FR‐FCM‐ZZNR). The original flow cytometry data from these previous studies were reprocessed with the same procedure as used in the current work.

### 2.3 TESTING FOR EVIDENCE AND IMPACTS OF SELECTION

We used the Brownian motion/Ornstein–Uhlenbeck framework to test for the presence of stabilizing selection on *trans*‐acting factors affecting *TDH3* promoter activity. We followed the approach of Bedford and Hartl (2009). Briefly, two models of quantitative trait evolution were fit to the observed expression values and phylogenetic relationships among strains. The Brownian motion model allowed for trait values to diverge linearly with time, while the Ornstein–Uhlenbeck model included an additional parameter that reflects the action of stabilizing selection. We tested whether the Ornstein–Uhlenbeck model fit significantly better than the Brownian motion model using a chi‐square distribution with a single degree of freedom.

In addition to this standard approach, we developed a method for identifying the action of natural selection on quantitative traits that uses empirically determined effects of new mutations to generate a neutral model of evolution for that specific trait. To inform this model, we used previously collected data on the effects on *TDH3* promoter activity due to new mutations. Briefly, this prior work used ethyl methanesulfonate (EMS) to induce mutations in an isogenic yeast population containing the P*_TDH3_*‐YFP reporter and used FACS to isolate ∼1500 individual genotypes irrespective of their YFP expression. Each isolated mutant contained ∼32 mutations relative to the reference strain, the overwhelming majority of which are expected to be *trans*‐acting with respect to *TDH3* promoter activity (only two mutations in the *TDH3* promoter are expected among all individuals; Metzger et al. 2016). To estimate how *TDH3* promoter activity could evolve in the absence of natural selection, we generated a neutral distribution by sampling effects from this mutational distribution, combining the effects of mutations multiplicatively. We repeated this process 10,000 times to create a distribution of effects on *TDH3* promoter activity expected under neutrality for a given number of mutations.

To test for natural selection, we compared the effects of changes in *TDH3* promoter activity due to *trans*‐regulatory differences among *S. cerevisiae* strains to our empirically derived neutral model. To account for the phylogenetic relationships among strains, we used a *S. cerevisiae* phylogeny estimated from whole genome polymorphism data to estimate how many mutations had likely occurred along each branch (MacLean et al. 2017). We estimated ancestral *TDH3 trans*‐regulation on the *S. cerevisiae* phylogeny using ACE and estimated likelihoods by simulation (Paradis et al. 2004). This approach was necessary because the neutral model of *TDH3* promoter activity evolution is based on nonnormally distributed data that preclude the assumption of time reversibility that allows for explicit calculation of the effects on expression on an unrooted phylogeny without identifying the direction of expression changes. We then compared the changes in *trans*‐regulatory effects along each branch to the corresponding distribution of effects derived from our neutral model. For each branch, we calculated the likelihood of the observed change in expression along that branch given the number of mutations that had occurred. We combined the likelihoods over all observed branches to determine the likelihood of the complete set of observed expression values and changes in expression on the phylogeny. Observed likelihoods less than expected under neutrality are consistent with positive selection for a new phenotypic value, whereas likelihoods greater than expected under neutrality are consistent with phenotypic constraint due to natural selection. Although there are biophysical limits on expression, the current modeling framework allows expression to increase or decrease without bounds. What these limits are and how they relate to the current expression level is unknown. Practically, as long as naturally occurring expression levels are not at a biophysical limit, natural selection can still be detected with this test. Because both increases and decreases in expression were observed among strains, new mutations, and segregants during mapping, this issue is unlikely to have substantially altered our conclusion.

The inference of ancestral states used for this test assumes that the expression value for each ancestral node is the average of the descendent node values weighted by the branch lengths. To test the robustness of our inference to phylogenetic uncertainty and this assumption of ancestral state values, we repeated the analysis using genetic distance among strains instead of phylogenetic branch lengths to estimate the number of mutations that had occurred among strains. To avoid double counting of individual strains, we used each strain exactly once in the comparison. We then sampled which strains were compared 10,000 times to generate a distribution of observed effects.

### 2.4 eQTL MAPPING

Genomic regions responsible for differences in *TDH3* promoter activity were identified by eQTL mapping. We crossed strains YPS1000 (PJW1057), SK1 (PJW1016), and M22 (PJW1072) that were MATalpha, nourseothricin resistant, and contained the P*_TDH3_*‐YFP reporter to a version of BY (PJW1240; Fig. S4) that was MATa, G418 resistant, and contained the P*_TDH3_*‐YFP reporter. This common BY mapping strain also contained a red fluorescent protein (RFP) marker at its mating type locus (Chin et al. 2012). Detailed methods for the creation of the common mapping strain are below. For each cross, we selected diploids using a combination of nourseothricin and G418 resistance and choose a single colony to ensure homogeneity in the genetic background. Hybrids for each cross were then sporulated (Fig. S2B, P.0). To do so, hybrids were grown on GNA (1% Difco yeast extract, 3% Difco nutrient broth, and 5% glucose) media for 12–16 h, transferred to KAc plates, and maintained at room temperature until at least 50% of the cells had sporulated. Cells were then washed twice in 1 mL of water and incubated with 200 µL of 0.3 mg/mL 100T zymolyase for 1 h with agitation. Next, cells were washed with 1 mL of water and resuspended in 100 µL of water. Cells were vortexed for 2 min to stick spores to the tube wall. The supernatant was removed and 1 mL of water was added. Without agitation, this 1 mL was removed and a second 1 mL of H_2_O was added. This 1 mL was also removed and 1 mL of triton‐X (0.02%) was added. Samples were sonicated on ice for 10 s at medium power (3.5 on a Sonic Dismembrator Model 100, Fisher). Spores were confirmed to be separated and diploids absent by visual inspection under a microscope.

After spore isolation, the population was split into thirds. One third was added to 1 mL YPD, grown to saturation overnight, and then frozen at –80°C as a glycerol stock. The second third was sorted for the absence of the RFP marker using FACS on a FACS canto II at the University of Michigan Flow Cytometry Core (Fig. S2B, P.1). Because all MAT**a** and diploid strains express RFP, this sorting captures only MATalpha cells. For each cross, we collected >10^6^ individuals lacking RFP fluorescence (Fig. S2B, F.1). These were incubated with 1 mL YPD and grown for 24–28 h. The final third was used to initiate additional rounds of crossing by plating onto YPD. After growth overnight, sporulation and spore isolation was repeated. Isolated spores from the second round of sporulation were plated onto YPD and sporulated a final time. After this third round of sporulation, cells lacking RFP fluorescence were again collected (Fig. S2B, F.3).

To identify the genetic basis of differences in YFP expression among strains, cells were transferred to 1 mL PBS and cells with the 5% highest (Fig. S2B, H.1.1 and H.3.1) or the 5% lowest (Fig. S2B, L.1.1 and L.3.1) YFP expression corrected for cell size were sorted from cells within the middle 80% of the cell size distribution based on forward scatter (FSC‐H). For each sample, 100,000 individuals were collected from each tail. Each sorted population was grown in liquid YPD for 20 h after which one half was frozen. To further enrich genotypes with high and low YFP expression within the sorted populations, the second half of each sample was used to initiate two additional rounds of sorting. For each round, the same sorting procedure as above was followed with one exception; populations originally sorted for high YFP expression were only sorted for high YFP expression and populations sorted for low YFP expression were only sorted for low YFP expression.

After all selection steps were completed, samples were revived from glycerol stocks and grown in 1 mL of YPD for 2 h. DNA was extracted from each sample using the Purgene Yeast Kit from Qiagen. DNA concentration was determined using a Qubit, and Illumina Nextera XT libraries were prepared following the manufacturers guidelines. Barcodes for each sample are listed in Table S2. Library quality was assessed using the bioanalzyer and all samples were pooled equally using concentration estimates from the Qubit. Sequencing was performed on a HiSeq 2000 using 125 bp paired end sequencing at the University of Michigan Sequencing Core. Sequencing barcodes are listed in Table S2.

### 2.5 QTL IDENTIFICATION

After sequencing, samples were processed to identify individual eQTL. First, Sickle was used to remove low‐quality bases from each read using default setting (Joshi and Fass 2011). Next, Cutadapt was used to remove any adapter sequence from read ends (flags ‐e 0.2 ‐O 3 ‐m 15; Martin 2011). Samples were aligned to the S228c reference genome using bowtie2 (flags ‐I 0 ‐X 1000 –very‐sensitive‐local; Langmead and Salzberg 2012) and then sorted and indexed using samtools (Li et al. 2009). Overlapping reads were clipped using clipOverlap in bamUtil. SNPs were jointly called within each paired set of samples selected for high and low YFP fluorescence using freebayes (Garrison and Marth 2012). Identified SNPs were required to reach at least 20% frequency in at least one of the two paired samples and be observed at least four times across both samples.

For each pair of samples, SNPs were filtered based on quality and depth. Each SNP was required to have depth of at least 20 to ensure adequate power, a depth below 500 to reduce the number of SNPs called in low complexity sequences, a mapping quality score of greater than 30, and imbalance scores for left/right, center/end, and forward/reverse for SNP position within reads of less than 30. At each position, only the two highest likelihood alleles were retained, with any other alleles observed assumed to result from sequencing errors. For each SNP, we calculated a G statistic using a likelihood ratio test of alternative and reference alleles within the high and low selected populations (Magwene et al. 2011). For SNPs where the alternative allele had a higher frequency than the reference allele in the high selected population relative to the low selected population, we maintained the sign of G. For SNPs where the alternative allele had a lower frequency than the reference allele in the high selected population relative to the low selected population, we flipped the sign of G. We then calculated G’ by averaging these estimates over a 40 kb window centered on each individual SNP using a tri‐cube kernel function (Magwene et al. 2011). To identify QTL peaks, we located all local maxima and minima in G’. We called significant peaks those with G’ > 5 or G’ < –5. The 95% confidence interval on the location of each peak was defined as the distance needed for G’ to drop by 5 from the peak. Local peaks whose confidence intervals overlapped in location were merged into a single peak. The G’ cutoff value was chosen to be conservative based on changes in SNP frequency from prior work (Magwene et al. 2011). We also compared allele frequency, G, and G’ for SNPs before and after sorting cells randomly with respect to YFP fluorescence for the M22 × BY cross. This experiment identified 11 eQTLs, giving a false discovery rate of approximately 10% for the mapping experiments reported in the main text. To determine how the specific G’ cutoff affects our analyses, we additionally conducted all analyses using a stricter G’ cutoff of 10, which corresponds to an FDR of 3%. Statistics for both cutoffs are included in Tables S5–S9.

### 2.6 CREATION OF MAPPING STRAIN

Determining the genetic and molecular mechanisms underlying complex phenotypes often requires identifying the causative genetic loci and nucleotides contributing to these traits (Rausher and Delph 2015). However, in the yeast *Saccharomyces cerevisiae*, the primary laboratory strains, S288c and its descendants, have several phenotypes that limit their usefulness in high throughput mapping approaches.

For example, *S. cerevisiae* isolates from the wild readily undergo meiosis under nutrient starvation and the majority of individual diploids sporulate. By contrast, S288c enters meiosis slowly and only a small proportion of individuals successfully complete meiosis, even under ideal conditions (Deutschbauer and Davis 2005; Gerke et al. 2006). Because genetic mapping requires recombination, and thus, meiosis, the limited meiotic abilities of S288c reduces the number and speed at which mapping populations can be created.

In addition to poor sporulation, S288c and its descendants generate petite cells lacking mitochondria with high frequency. As a consequence, these individuals cannot perform aerobic respiration and often have altered phenotypes compared to wild‐type individuals (Chen and Clark‐Walker 1999). Because linking phenotypes to their genomic location requires high‐quality phenotyping, additional variation introduced by petite individuals can reduce the accuracy and power of genetic mapping.

Finally, upon meiosis yeast generate both **a** and α haploids. These haploids will readily reform diploids if not prevented, thus introducing additional variation due to ploidy into a mapping population. Current techniques for limiting the recreation of diploids suffer from a lack of throughput and poor specificity (Tong et al. 2001). As a consequence, the power to map the genetic basis of recessive traits in yeast is reduced.

To overcome these deficiencies, we modified S288c to increase its sporulation rate and density, reduce the frequency at which it generated petites, and to express a fluorescent marker that allowed easy identification of mating type. To accomplish these goals, we obtained several strains derived from S288c. These strains vary in their mating type and auxotrophies, facilitating crossing. In addition, these strains differ at a set of alleles derived from natural *S. cerevisiae* strains that either improve sporulation rate or lower petite frequency. These include versions of *TAO3* and *RME1* that increase sporulation rate (Deutschbauer and Davis 2005) and versions of *SAL1*, *CAT5*, and *MIP1* that decrease petite frequency (Dimitrov et al. 2009). An allelic variant at *MKT1* has also been identified that affects both sporulation and petite frequency. However, while the wild‐type S288c MKT1 allele decreases sporulation rate, it also substantially reduces petite frequency compared to the alternative allele and we kept the S288c version (Deutschbauer and Davis 2005; Dimitrov et al. 2009).

Through a series of crosses, transformations, and sporulations, we isolated a single individual that contained the desired set of alleles and was free of all auxotrophies except for ura3Δ0 (Fig. S4). We retained the *URA3* auxotrophy to facilitate future genetic manipulation by the *delitto perfetto* method, which requires *5‐FOA* counter‐selection and therefore a starting strain that is ura‐ (Storici and Resnick 2006). To facilitate the creation of the correct strain, we tracked the allelic identity of each segregating locus using pyrosequencing (Tables S3 and S4). After identification, the isolated individual was turned into a diploid and sporulated to generate isogenic **a** and α haploids. To the **a** haploid, we introduced a red fluorescent protein into the *MAT* locus (Chin et al. 2012). This marker allows identification of individuals based on their mating type using FACS. Using this marker, populations containing millions of individuals of the same mating type can be collected in minutes. Finally, both **a** and α strains contain a *TDH3* promoter driving YFP expression located at the *HO* gene to facilitate mapping of mutations and polymorphisms influencing *TDH3* expression.

Growth was performed using YPD. For crosses involving auxotrophies, synthetic complete media was used, minus the appropriate amino acids (1.7 g yeast nitrogen base, 5 g ammonium sulfate, 20 g glucose per 1 L water; 20 g agar for solid plates). Sporulation was induced by growth on YPD plates for 24 h at room temperature, followed by plating on KAc plates at room temperature (10 g potassium acetate, 0.5 g glucose per 1 L water; 20g agar for solid plates). Ascus walls were dissolved prior to tetrad dissections by incubating spores in 200 µL zymolyase (1 mg/mL 20T) for 1 h without shaking.

To create homozygous diploids from haploids, strains were transformed with plasmid pCM66. pCM66 contains a galactose inducible copy of *HO* and a selective nourseothricin resistance marker. After transformation, nourseothricin resistant cells were grown with galactose as the sole carbon source at 30°C without shaking for 8 h to induce expression of *HO*. This allowed for mating type switching and subsequent mother–daughter cell mating to produce diploids. Cells were streaked for single colonies on YPD plates and the ploidy of single colonies checked by colony PCR using mating‐type specific primers. Diploid colonies were streaked onto fresh, nonselective, YPD plates and assayed for loss of nourseothricin resistance, and thus pCM66.

Pyrosequencing was used to follow sporulation and petite QTN. Methods are as described in Wittkopp (2012). PCR primers used are Table S3. Dispensation order for pyrosequencing is in Table S4.

## 6 CONFLICT OF INTEREST

The authors declare no conflicts of interest.

7

Associate Editor: S. Wright

## Supporting information


**Figure S1**. Sources of variation in *TDH3* expression and tests for selection.
**Figure S2**. Mapping eQTL affecting TDH3 expression.
**Figure S3**. eQTL identified with alternative mapping strategies.
**Figure S4**. Creation of common mapping strain PJW1240.
**Table S1**. Natural strains used for *trans*‐regulatory polymorphism effects.
**Table S2**. Barcode sequences used for *TDH3 trans*‐regulatory eQTL mapping.
**Table S3**. Primer sequences for tracking sporulation and petite QTN by pyrosequencing.
**Table S4**. Sequences and dispensation order for pyrosequencing.
**Table S5**. eQTL identified using a FDR of 10%.
**Table S6**. eQTL identified using a FDR of 3%.
**Table S7**. eQTL Overlap Statistics using a FDR of 10%.
**Table S8**. eQTL Overlap Statistics using a FDR of 3%.
**Table S9**. Overlap of second M22 x BY experiment using a FDR of 10%.
**Table S10**. Overlap of second M22 x BY experiment using a FDR of 3%.
**Table S11**. eQTL location and direction of effect (FDR of 10%).
